# Biodiversity policy beyond economic growth

**DOI:** 10.1111/conl.12713

**Published:** 2020-04-13

**Authors:** Iago Otero, Katharine N. Farrell, Salvador Pueyo, Giorgos Kallis, Laura Kehoe, Helmut Haberl, Christoph Plutzar, Peter Hobson, Jaime García‐Márquez, Beatriz Rodríguez‐Labajos, Jean‐Louis Martin, Karl‐Heinz Erb, Stefan Schindler, Jonas Nielsen, Teuta Skorin, Josef Settele, Franz Essl, Erik Gómez‐Baggethun, Lluís Brotons, Wolfgang Rabitsch, François Schneider, Guy Pe'er

**Affiliations:** ^1^ Integrative Research Institute on Transformations of Human‐Environment Systems (IRI THESys) Humboldt‐Universität zu Berlin Berlin Germany; ^2^ Interdisciplinary Centre for Mountain Research University of Lausanne Lausanne Switzerland; ^3^ Biology Program, Faculty of Natural Sciences Universidad del Rosario Bogotá Colombia; ^4^ Berlin Workshop in Institutional Analysis of Social‐Ecological Systems Humboldt‐Universität zu Berlin Berlin Germany; ^5^ Research & Degrowth Barcelona Spain; ^6^ Department of Evolutionary Biology, Ecology and Environmental Sciences Universitat de Barcelona Catalonia Spain; ^7^ Institute of Environmental Science and Technology (ICTA) Autonomous University of Barcelona Barcelona Spain; ^8^ ICREA Barcelona Spain; ^9^ Geography Department Humboldt‐Universität zu Berlin Berlin Germany; ^10^ Biology Department University of Victoria Victoria Canada; ^11^ Department of Forest & Conservation Sciences University of British Columbia Vancouver Canada; ^12^ The Nature Conservancy London UK; ^13^ Oxford Martin School University of Oxford Oxford UK; ^14^ Institute of Social Ecology University of Natural Resources and Life Sciences Vienna Austria; ^15^ Division of Conservation Biology, Vegetation Ecology and Landscape Ecology, Department of Botany and Biodiversity Research University of Vienna Vienna Austria; ^16^ Centre for Econics & Ecosystem Management Writtle University College Chelmsford UK; ^17^ Leibniz‐Institute of Freshwater Ecology and Inland Fisheries Berlin Germany; ^18^ Energy and Resources Group University of California Berkeley Berkeley United States; ^19^ Centre d’Écologie Fonctionnelle et Évolutive UMR 5175, CNRS–Université de Montpellier–Université Paul Valéry Montpellier–École Pratique des Hautes Études IRD Montpellier France; ^20^ Community Ecology and Conservation research group, Faculty of Environmental Sciences Czech University of Life Sciences Prague Czech Republic; ^21^ Freelance biodiversity conservationist Zagreb Croatia; ^22^ German Centre for Integrative Biodiversity Research (iDiv) Halle‐Jena‐Leipzig Leipzig Germany; ^23^ UFZ ‐ Helmholtz Centre for Environmental Research Department of Community Ecology Halle Germany; ^24^ Institute of Biological Sciences University of the Philippines Los Baños College Laguna Philippines; ^25^ Department of International Environment and Development Studies (Noragric) Norwegian University of Life Sciences (NMBU) Ås Norway; ^26^ Norwegian Institute for Nature Research (NINA) Oslo Norway; ^27^ InForest Joint Research Unit (CTFC‐CREAF) Solsona Spain; ^28^ CREAF Cerdanyola del Vallès Spain; ^29^ CSIC Cerdanyola del Vallès Spain; ^30^ Environment Agency Austria Vienna Austria; ^31^ Research & Degrowth France Cerbère France; ^32^ UFZ ‐ Helmholtz Centre for Environmental Research Department of Ecosystem Services and Department of Environmental Economics Leipzig Germany; ^33^ University of Leipzig Leipzig Germany

**Keywords:** biodiversity conservation, biodiversity loss, biodiversity policy, biodiversity scenarios, decoupling, degrowth, economic growth, postgrowth, sustainability policy, transition

## Abstract

Increasing evidence—synthesized in this paper—shows that economic growth contributes to biodiversity loss via greater resource consumption and higher emissions. Nonetheless, a review of international biodiversity and sustainability policies shows that the majority advocate economic growth. Since improvements in resource use efficiency have so far not allowed for absolute global reductions in resource use and pollution, we question the support for economic growth in these policies, where inadequate attention is paid to the question of how growth can be decoupled from biodiversity loss. Drawing on the literature about alternatives to economic growth, we explore this contradiction and suggest ways forward to halt global biodiversity decline. These include policy proposals to move beyond the growth paradigm while enhancing overall prosperity, which can be implemented by combining top‐down and bottom‐up governance across scales. Finally, we call the attention of researchers and policy makers to two immediate steps: acknowledge the conflict between economic growth and biodiversity conservation in future policies; and explore socioeconomic trajectories beyond economic growth in the next generation of biodiversity scenarios.

## 1 INTRODUCTION

Conservation scientists have long stressed the need to pay attention to the socioeconomic context of biodiversity loss if effective policies are to be designed (Martin, Maris, & Simberloff, 2016). Such a question becomes urgent in the face of an unprecedented degradation of the biosphere, undermining human well‐being and calling into question the standard development model (IPBES, 2019a). As economic growth is part and parcel of this development model (Escobar, 2015), the exploration of its effects on biodiversity has the potential to strengthen the diagnosis of biodiversity decline and support the design of effective solutions.

The critical assessment of economic growth has recently been directly linked to the debates around biodiversity conservation. Authors have highlighted the need to move away from the global economy's current foundation on economic growth while discussing the role of conservation science in the transition to a society focused instead on biodiversity and well‐being (Büscher et al., 2017; Martin et al., 2016; see also Czech, Krausman, & Devers, 2000). However, why and how a critical assessment of economic growth may improve biodiversity policies in an ambitious and yet realistic way remains unexplored.

This paper aims to shed light on this crucial question. To do so, we first synthesize available empirical evidence on the relationships between economic growth and biodiversity, focusing on land‐use change, climate change, and invasive alien species. Second, we review the prospects for decoupling economic growth from biodiversity loss. Third, we review the position of 28 international biodiversity and sustainability policy documents (produced under the auspices of the United Nations between 1972 and 2016) about economic growth and decoupling. Fourth, we sketch out policy possibilities by presenting existing literature on alternatives to economic growth and reviewing its relevance for biodiversity conservation. Finally, we show how scenario development for major policy instruments, such as the Convention on Biological Diversity, could help directing national and international priorities away from the growth imperative and toward the enhancement of biodiversity and human well‐being.

## 2 ECONOMIC GROWTH, RESOURCE USE, AND BIODIVERSITY LOSS

**FIGURE 1:**
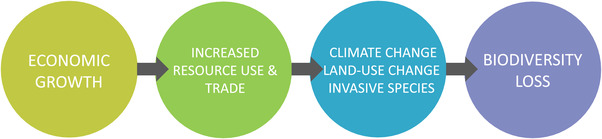
How economic growth contributes to biodiversity loss. Economic growth increases resource use and trade, which in turn impact biodiversity via various mechanisms reviewed in the text (climate change, land‐use change, and invasive species). Source: our own

Increasing evidence shows that an expanding economy degrades biodiversity. In this paper, biodiversity is understood as the variability among living organisms and the ecological complexes of which they are a part. This can include variation in genetic, phenotypic, phylogenetic, and functional attributes, as well as changes in abundance and distribution over time and space, within and among species and ecosystems (IPBES, 2019a, glossary). The connection between economic growth and biodiversity loss can be explored by resorting to correlations between gross domestic product (GDP), resource use and the state of biodiversity (Figure 1). While such correlations do not necessarily imply causality, the arguments assembled below suggest that causal relations do exist. Next, we show the relevance of our rationale for three well‐known mechanisms of biodiversity loss.

### 2.1 Land‐use change

Global agricultural area has increased by ca. 70–80% during the twentieth century, and agricultural production has increased nearly sixfold as a result of land‐use intensification (Klein Goldewijk, Beusen, Doelman, & Stehfest, 2017; Krausmann et al., 2013). This increasing—and increasingly intense—use of land for agriculture has been attributed to different drivers such as population, yield, and diet (Alexander et al., 2015). The structure and evolution of the global economy seem to play a key role though. Trends in global agricultural land and in fertilizer and pesticide use correlate with GDP since the 1960s (Tilman et al., 2001). Increases in per‐capita GDP also closely correlate with a higher demand for animal protein (Tilman & Clark, 2014), which further increases the demand for agricultural area (Alexander et al., 2015; Kastner, Rivas, Koch, & Nonhebel, 2012).

GDP growth is also associated with an expansion of urban areas and infrastructures (Seto, Güneralp, & Hutyra, 2012). The total mass of global human‐made material stocks (buildings, roads, etc.) grew in unison with global GDP over the last century, replacing ecosystems at a massive scale (Krausmann et al., 2017).

Agricultural expansion and the development of cities and infrastructures threaten biodiversity through the encroachment and fragmentation of habitats, both major causes of biodiversity loss across almost all terrestrial taxonomic groups (Andrén, 1994; Didham, Ghazoul, Stork, & Davis, 1996; Fischer & Lindenmayer, 2007; Krauss et al., 2010). Conventional agricultural intensification—characterized by a shift to highly mechanized, large‐scale monocultures with high levels of agrichemicals use—is often detrimental to biodiversity (Newbold et al., 2015). These intensification processes can increase the risk of soil erosion, degradation (Foucher et al., 2014; IPBES, 2018a), and salinization (Foresight, 2011). They can also reduce soil organic matter, disturb soil biota communities (Foucher et al., 2014; Postma‐Blaauw, de Goede, Bloem, Faber, & Brussaard, 2010), result in biotic homogenization, become toxic to plants with cascading effects on ecosystems (Yamaguchi & Blumwald, 2005), and threaten birds, mammals, amphibians, and insects (Gibbs, Mackey, & Currie, 2009; Hof, Araújo, Jetz, & Rahbek, 2011; IPBES, 2017; Kerr & Cihlar, 2004; Kleijn et al., 2009).

### 2.2 Climate change

Global economic and population growth have driven an increase in anthropogenic greenhouse gas (GHG) emissions, leading to unprecedented atmospheric concentrations that have warmed the climate (IPCC, 2014). Global carbon dioxide and other GHG emissions increase with GDP, and there is no empirical evidence for the assumption that they would automatically start declining in absolute terms once a certain threshold of GDP has been reached (Burke, Shahiduzzaman, & Stern, 2015; Stern, 2017; for national‐level decoupling, see Section 3).

Shifts toward warmer climates are occurring at an unprecedented rate that may exceed the capacity of many species and ecosystems to adapt, leading to changes in species ranges and population sizes, and resulting in local extinctions (Burrows et al., 2011; Hof et al., 2011; Wessely et al., 2017). Warmer temperatures have affected the phenology and the distribution of species from various taxonomic groups across the globe (Cohen, Lajeunesse, & Rohr, 2018; Parmesan & Yohe, 2003; Peñuelas & Filella, 2001; Root et al., 2003; Walther et al., 2002). Breeding bird populations, for instance, have shown a consistent response to climate change across the United States and Europe since 1980, with an increasingly divergent fate between species favored and disadvantaged by rising temperatures (Stephens et al., 2016). Between 2001 and 2008, European mountain plant communities experienced a decline in cold‐adapted species and an increase in warm‐adapted ones, as well as upward shifts in ranges (Gottfried et al., 2012; Pauli et al., 2012). The northward shift of European bird and butterfly communities observed between 1990 and 2008 was insufficient to track temperature changes (Devictor et al., 2012).

In addition, climate change modifies habitats and enhances the frequency and intensity of extreme events such as storms, floods, extreme temperatures, and droughts (Maxwell, Fuller, Brooks, & Watson, 2016). In fact, extreme events are considered to pose an even greater threat to biodiversity than global warming, both in terrestrial and marine environments (Garcia, Cabeza, Rahbek, & Araújo, 2014; Wernberg et al., 2013). Moreover, changes in climate can undermine efforts to conserve biodiversity: for instance, in Europe 58% of plant and terrestrial vertebrate species are projected to lose suitable climate conditions within existing protected areas by 2080 (Araújo, Alagador, Cabeza, Nogués‐Bravo, & Thuiller, 2011). Climate change could also cause abrupt system‐level shifts in several biomes on the earth (Lenton, 2013). Finally, the effects of climate change are likely to act in synergy with the effects of land‐use change, especially as species’ dispersal and adaptation to changing conditions is hindered by habitat loss and fragmentation (Sirami et al., 2017; Urban et al., 2016).

### 2.3 Invasive alien species

Economic growth is intimately related to international trade and the expansion of transport routes (Dittrich & Bringezu 2010; Schandl et al., 2017). In turn, international trade provides numerous opportunities for the transport of propagules of alien species to new regions (Seebens et al., 2015). Seebens et al. (2015) show that a strong increase in alien plant species is expected in the next decades, especially for emerging economies in megadiverse regions.

The human‐caused introduction and spread of species in regions that were previously beyond the reach of natural colonization has become a defining feature of global biodiversity loss (IPBES, 2019a). Alien species are the second most common threat associated with the extinction of plants, amphibians, reptiles, birds, and mammals (Bellard, Cassey, & Blackburn, 2016). Impacts of alien species are particularly pronounced on islands, where evolutionary naïve native species often become exposed to novel predators, pathogens, or strong competitors. Accordingly, 86% of documented historic extinctions on islands are linked to biological invasions (Bellard et al., 2016). An unprecedented intensity of human‐mediated species exchange is associated with contemporary economic activities, leading to the homogenization of flora and fauna (Capinha, Essl, Seebens, Moser, & Pereira, 2015; Winter et al., 2009), redefining the classical boundaries of biogeography (Capinha et al., 2015), and presenting far‐reaching negative implications for native biota, ecosystem services, and human well‐being (Vilà & Hulme, 2017; Vilà et al., 2010).

As the global economy grows, the increase in the numbers of alien species does not show any sign of saturation (Seebens et al., 2017). Thus, many new introductions and associated negative impacts can be expected in the future (Seebens et al., 2018).

## 3 DECOUPLING ECONOMIC GROWTH FROM BIODIVERSITY LOSS?

In theory, increases in the efficiency of resource use could enable economic growth while reducing environmental and biodiversity impacts. This possibility is referred to as *decoupling*. *Relative decoupling* means that GDP grows faster than resource use. It has been observed in the global aggregate as well as in many countries over long (decadal) periods of time for measures of aggregate use of resources (materials and energy) and GHG emissions in the last century (Haberl et al., 2019). *Absolute decoupling* means that resource use declines in absolute terms while GDP grows; this requires that resource efficiency (i.e., the ratio GDP/resource use) grows faster than GDP. The literature has provided ample evidence that sustained absolute decoupling has not occurred so far (Alexander et al., 2015; Csereklyei & Stern, 2015; Krausmann et al., 2013; Steinberger, Krausmann, Getzner, Schandl, & West, 2013; Ward et al., 2016; Wiedmann et al., 2015; see below for some nuances). These studies suggest that, under current socioecological conditions, economies with higher GDP tend to (i) consume more raw materials and energy, (ii) occupy more productive land, and/or (iii) use it more intensively.

With regard to raw materials, a panel analysis of 39 countries (1970–2005) found that a 1% growth in GDP per capita implied a 0.8% growth in material use per capita (Steinberger et al., 2013). Krausmann, Schandl, Eisenmenger, Giljum, and Jackson (2017) found that global relative decoupling of materials from GDP ground to a halt around 2002; thereafter, global material productivity (GDP/material use) deteriorated due to growth in regions with resource‐intensive production such as China. The few cases of absolute decoupling they found were related to low GDP growth and to increased import of material‐intensive goods. Similarly, the domestic material use of some countries in the Global North declined in absolute terms while their economies grew (1990–2008), but this was achieved by importing resource‐intensive goods from the Global South (Wiedmann et al., 2015). When all raw materials associated with imported and exported goods are considered, the material footprint of these countries increases with GDP, although not at the same rate (Wiedmann et al., 2015).

In the case of the human appropriation of net primary production (HANPP), global data show a (strong) relative decoupling. In the period 1910–2005, global GDP increased much faster than global HANPP (17‐fold vs. twofold) (Krausmann et al., 2013). However, this was due to (i) land‐use intensification, which resulted in NPP increases and partly compensated for growing harvest volumes, and (ii) most non‐land‐use–based economic activity being reliant on fossil energy and not biomass (Krausmann et al., 2013). As noted above, both land‐use intensification and fossil energy use (climate change) impact biodiversity, suggesting that this relative decoupling can have considerable trade‐offs for biodiversity conservation.

Regarding CO_2_ emissions, a steady increase is observed at the global level for the period 1960–2018 (Global Carbon Budget, 2018). An analysis of 189 countries for the period 1961–2010 found that a 1% increase in GDP was associated with a 0.5–0.8% increase in CO_2_ emissions (Burke et al., 2015). In the period 2006–2016, the United States and EU28 had declining emissions in absolute terms despite continued economic growth, in both territorial and consumption‐based terms (Global Carbon Budget 2018; see also Quéré et al., 2019). These results indicate that absolute decoupling could be possible. However, these declines are far slower than those needed to meet the 1.5°C Paris target (Hickel & Kallis, 2019).

In the case of biodiversity, an absolute decoupling between economic growth and impacts occurred in Western Europe and North America during the period 2000–2011, considering both production and consumption (Marques et al., 2019). As these authors show, this decoupling was associated with a reduction in consumption following the financial crisis, after which biodiversity impacts increased again. At the global level and in the same period, despite a reduction of biodiversity impacts per unit of GDP, overall population and economic growth resulted in increased total impacts (Marques et al., 2019).

Some studies suggest that absolute decoupling could be possible in the future under scenarios of dramatic reductions in energy demand through highly efficient technologies and structures (Grubler et al., 2018). Yet other studies argue that absolute decoupling is unlikely to occur, specially at a fast enough rate to ensure global sustainability (Hickel & Kallis, 2019; Jackson & Victor, 2019; Ward et al., 2016).

The possibility of absolute decoupling is implicitly defended through reference to the environmental Kuznets curve (EKC). The EKC applied to biodiversity predicts that biodiversity damage first increases and then decreases with rising per capita incomes, as higher levels of income bring about demand for, and investment in, biodiversity conservation (Dietz & Adger, 2003). Partial support for a biodiversity EKC has only been found for threatened bird and mammal species in two multicountry analyses (McPherson & Nieswiadomy, 2005; Naidoo & Adamowicz, 2001), as well as for birds linked to some habitat types in a number of Canadian provinces (Lantz & Martínez‐Espiñeira, 2008). However, several multicountry analyses found no evidence to support an EKC effect for a range of terrestrial and aquatic biodiversity proxies, even supporting a trend in the opposite direction from that predicted by the EKC hypothesis (Clausen & York, 2008; Dietz & Adger, 2003; Gren, Campos, & Gustafsson, 2016; Majumder, Berrens, & Bohara, 2006; Mills & Waite, 2009).

In the United States, the existence of an EKC was not supported for an integrated index of biodiversity risk (Tevie, Grimsrud, & Berrens, 2011). In this country, avian biodiversity was found to follow an S‐curve relationship, rather than the U‐curve of the EKC—that is, biodiversity initially declines with economic growth, then improves over intermediate growth, and ultimately declines at higher growth (Strong, Tschirhart, & Finnoff, 2011). These observations resonate with other studies carried out in the United States indicating a close link between GDP growth and species endangerment (Czech et al., 2005; Czech, Mills Busa, & Brown, 2012), and with theoretical analyses arguing that economic growth results in the competitive exclusion of nonhuman beings (Czech, 2008). Given the evidence assembled in this paper, such a close link is unlikely to be a coincidence.

## 4 ECONOMIC GROWTH AND DECOUPLING IN INTERNATIONAL SUSTAINABILITY AND BIODIVERSITY POLICIES

Advocacy of economic growth is unequivocal in some of the most influential policy documents on sustainability and biodiversity analyzed in this paper (see selection criteria in SM1). The first major international declaration concerning sustainable development, the 1987 *Brundtland* report, called for “internationally expansionary policies of growth” in industrial countries and for “more rapid economic growth in both industrial and developing countries”.^1^ This commitment has since been reiterated in all subsequent major sustainability declarations and agreements (Gómez‐Baggethun & Naredo, 2015). The Declaration of the UN Conference on Environment and Development held in Rio de Janeiro in 1992 advocated “economic growth and sustainable development in all countries, to better address the problems of environmental degradation”^2^; the 2011 UN Environment Programme (UNEP) report on the green economy stated that “the key aim for a transition to a green economy is to enable economic growth and investment while increasing environmental quality”^3^; and the Rio 2012 declaration reaffirmed “the need to achieve sustainable development by promoting sustained, inclusive and equitable economic growth”.^4^ The current UN Sustainable Development Goals likewise call for “sustainable economic growth” and to “sustain per capita economic growth”.^5^ In keeping with this trend, the declaration of the Cancun Conference of the Parties to the Convention on Biological Diversity (CBD) commits signatories to “promote sustainable economic growth”.^6^

While advocating economic growth, key policy documents on sustainability and biodiversity conservation acknowledge the relevance of drivers of biodiversity loss that are strongly related to economic growth according to the review presented in Section 2 (Table S4 in the Supporting Information). Indeed, their views on the relationship between economic growth and biodiversity are mostly ambiguous, and very few of them (six out of 28) explicitly recognize that growth is problematic for biodiversity (Tables 1 and 2). More than half of these documents (16) neglect the question of how a decoupling of economic growth from biodiversity loss might be achieved. Among those that do address this question (12), only seven accept that reducing the pressures of a growing economy on biodiversity is challenging (Tables 1 and 2). This is the case, for example, of the Global Biodiversity Outlook 4, which explicitly recognizes that absolute decoupling is unlikely given current patterns of consumption^7^. The other documents that do address the question of decoupling either have ambiguous positions or consider it to be unchallenging. The latter is the case of the Cancun declaration, which limits itself to listing several measures to reduce the biodiversity impacts of economic growth, without recourse to a scientific assessment of their success prospects within the current economic system.

**TABLE 1:** Policy analysis: How key international declarations and agreements on sustainability and biodiversity view the relationship between economic growth and biodiversity, and how they view the prospects of decoupling economic growth from biodiversity loss

		A	B	C
	Document	View on the relationship between economic growth and biodiversity	Is decoupling mentioned?	View on decoupling economic growth from biodiversity loss
Policy documents on sustainability	Declaration UN Conference on the Human Environment Stockholm (1972)	Problematic	Yes	Challenging
	UN Report of the World Commission on Environment and Development (1987) (Brundtland Report)	Ambiguous	Yes	Challenging
	Declaration UN Conference on Environment and Development Rio de Janeiro (1992)	Ambiguous	No	NA
	Declaration UN World Summit on Sustainable Development Johannesburg (2002)	Unproblematic	Yes	Unchallenging
	Millennium Ecosystem Assessment (2005)	Ambiguous	Yes	Challenging
	Declaration UN Conference on Sustainable Development Rio de Janeiro (2012) (Rio + 20)	Problematic	No	NA
	UN Sustainable Development Goals (2015)	Ambiguous	Yes	Unchallenging
Policy documents on biodiversity	Convention on Biological Diversity (1992)	Ambiguous	No	NA
	Report CBD COP 1 (1994)	Ambiguous	No	NA
	Report CBD COP 2 (1995)	Problematic	No	NA
	Report CBD COP 3 (1996)	Ambiguous	Yes	Challenging
	Report CBD COP 4 (1998)	Ambiguous	Yes	Challenging
	Report CBD COP 5 (2000)	Ambiguous	No	NA
	Cartagena Protocol on Biosafety to the CBD (2000)	Unproblematic	No	NA
	Report CBD COP 6 (2002)	Problematic	No	NA
	Report CBD COP 7 (2004)	Unproblematic	No	NA
	Report CBD COP 8 (2006)	Ambiguous	Yes	Challenging
	Report CBD COP 9 (2008)	Ambiguous	No	NA
	Report CBD COP 10 (2010)	Ambiguous	Yes	Ambiguous
	Strategic Plan 2011–2020 and Aichi Targets CBD COP 10 (2010)	Unproblematic	No	NA
	Nagoya ‐ Kuala Lumpur Supplementary Protocol to Cartagena Protocol (2011)	Ambiguous	No	NA
	Nagoya Protocol on Access to Genetic Resources to the CBD (2011)	Unproblematic	No	NA
	Report CBD COP 11 (2012)	Problematic	Yes	Ambiguous
	Report CBD COP 12 (2014)	Ambiguous	No	NA
	Gangwon Declaration CBD COP 12 (2014)	NA	No	NA
	Global Biodiversity Outlook 4 (2014)	Problematic	Yes	Challenging
	Opening statement to CBD COP 13 (2016)	NA	No	NA
	Cancun Declaration CBD COP 13 (2016)	Ambiguous	Yes	Unchallenging

*Note*: Column A. “Problematic”: Growth is explicitly presumed to have either a negative, or potentially negative, impact on biodiversity. “Unproblematic”: Growth is explicitly presumed to have either no impact or a positive impact on biodiversity. “Ambiguous”: The position is either internally contradictory, sometimes seen as problematic sometimes not, or too vague to be determined. “NA”: Not assessed. Column C. “Challenging”: Decoupling economic growth from biodiversity loss is explicitly presumed to be complicated, difficult, or potentially impossible. “Unchallenging”: Decoupling economic growth from biodiversity loss is explicitly presumed to be easy and/or automatic. “Ambiguous”: The position on decoupling is either internally contradictory, sometimes seen as problematic sometimes not, or too vague to be determined. “NA”: The relationship was not assessed if the document did not mention decoupling. For methods and full results of the review of policy documents, see SM1.

**TABLE 2:** Summary of results from policy analysis (Table 1)

	A. View on the relationship between economic growth and biodiversity				B. Is decoupling mentioned?		C. View on decoupling economic growth from biodiversity loss			
	Problematic	Unproblematic	Ambiguous	NA	Yes	No	Challenging	Unchallenging	Ambiguous	NA
Policy documents on sustainability (7)	2	1	4	0	5	2	3	2	0	2
Policy documents on biodiversity (21)	4	4	11	2	7	14	4	1	2	14

Other key biodiversity policies do not acknowledge the problematic nature of economic growth at all when addressing drivers of biodiversity loss. For instance, the CBD Aichi Targets for 2020 aimed to contain “the impacts of use of natural resources well within safe ecological limits”,^8^ without addressing the systemic relationships between economic growth and the critical global biodiversity pressures shown to undermine progress toward the targets. These pressures include ecological and water footprints, trawl fishing effort, nitrogen surplus, and introduction of alien species (Tittensor et al., 2014). This means that several Aichi targets (and future similar targets) may be unachievable unless clear progress is made in explicitly addressing the impacts of economic growth.

In light of ample evidence showing that absolute decoupling is unlikely under current conditions, the unreflexive growth emphasis of the biodiversity and sustainability policies seems to stand in the way of safeguarding biodiversity.

## 5 BIODIVERSITY AND ECONOMIC POLICIES BEYOND ECONOMIC GROWTH: SOLUTIONS AND CHALLENGES

Biodiversity policies reflect the shared assumption by policy‐makers that economic growth is needed to alleviate poverty and to achieve prosperity (Table S4 in SM1). However, an emerging literature explores whether and how it may be possible to find a “prosperous way down” and manage without growth (D'Alisa, Demaria, & Kallis, 2014; Daly, 1991; Jackson, 2011; Odum & Odum, 2006; Victor, 2008). This literature has its origins in the Global North, where strategies for alternative economies thrive on an intellectual and material history that is far from that of the Global South. Yet analogous values—such as subsistence‐living, balance between all living beings, and reciprocity—favor a joint exploration of alliances (Escobar, 2015; Rodríguez‐Labajos et al., 2019).

This literature—composed of different schools—argues that policy‐makers can design policies to control unsustainable expansion. Steady‐state economics proposes legal limits to throughput (the economy´s use of energy and materials), allowing the economy to develop qualitatively within such limits (Daly, 1996; Dietz & O'Neill, 2013). Degrowth scholars call for abolishing the pursuit of GDP growth and highlight the potential of grassroots movements for facilitating the transition to a new economy (Kallis, 2011; Kallis et al., 2018). Whereas the degrowth literature considers a reduction of GDP inevitable if throughput is to decrease to sustainable levels, the postgrowth literature prefers to ignore GDP, which is deemed a bad indicator of welfare, and argues for environmental and well‐being policies, regardless of their effects on GDP (Raworth, 2017; van den Bergh & Kallis, 2012).

Policy proposals from this literature can contribute to reframing biodiversity and economic policies beyond the economic growth imperative (Table S5 in SM2), even if remarkable challenges are to be expected (Box 1). The establishment—via multilevel governance—of absolute caps on the amount of resources embedded in imported goods and services is crucial (Alcott, 2010; Daly, 1991). Different caps could apply to different countries depending on their past consumption and ecological or carbon debts (Martinez‐Alier, 2002). Caps could be complemented by specific moratoria on resource extraction in highly sensitive biodiverse regions—so‐called “resource sanctuaries” (Videira, Schneider, Sekulova, & Kallis, 2014)—and by limiting the expansion of large infrastructures, which not only enhance the extractive capacity of nations (Krausmann et al., 2017) but also represent a direct threat to biodiversity (Ibisch et al., 2016; Maxwell et al., 2016; Table S5 in SM2). The global map of roadless areas is a cost‐effective means for guiding this endeavor, as it highlights the potential of, and the urgent need for, protecting key biodiversity refugia from road expansion (Ibisch et al., 2016).

BOX 1 **CHALLENGES OF IMPLEMENTING BIODIVERSITY POLICIES BEYOND ECONOMIC GROWTH. SOURCE: OUR OWN**

Measures such as a reduction of working hours and resource caps may benefit biodiversity, but their implementation faces several challenges. *Social and cultural barriers* are to be expected since voluntary simplicity goes against the prevalent imaginary of unlimited growth. However, evidence suggests that the desire for more personal time, environmental and ethical factors, and health reasons motivate people to seek a simpler life, mostly by working less (Alexander & Ussher, 2012). The greatest obstacle for this is the structural incentive to overwork. Moreover, modern societies require material growth in order to preserve the socioeconomic and political status quo (Rosa, Dörre, & Lessenich, 2017). Therefore, calls to go beyond economic growth in biodiversity policies will also find *political and legal barrier*s. By questioning the assumption that economic growth is necessary to ensure prosperity, such calls aim at “repoliticizing” the sustainability debate (Asara, Otero, Demaria, & Corbera, 2015). The political confrontation between alternative societal models can be an opportunity to expand the solutions space for biodiversity conservation. Whether alternative ideas will permeate national and international legal frameworks influencing the planet's biodiversity will ultimately depend on the ability of political actors to forge new consensus beyond economic growth. Finally, *corporate barriers* should not be neglected. Industries tend to endorse policy initiatives that secure growing access to resources from global markets, thus against the rationale of resource caps. The European Union's Raw Materials Initiative is a good example of this (European Commission, 2019). Furthermore, revenue is a basic driver of corporate profit. Faced with societal and political decisions for reduced resource use, companies may generate signals that act as disincentives for further resource savings. For instance, when domestic water usage in Barcelona dropped to less than 120 liters per person per day by 2008 (Tello & Ostos, 2012), the average billing increased by 60% in the following 5 years. The private company in charge of domestic water supply provided economic viability reasons to justify the fee increases (Cordero, 2013).

When designing policies for a prosperous way down, one core concern is what would happen to employment. Lack of growth in growth‐based economies increases unemployment and causes instability. But high unemployment is not a necessary outcome of an economic slowdown: 1% less growth in Japan or Austria leads to only 0.15% more unemployment, compared to 0.85% in Spain (Ball, Leigh, & Loungani, 2013). Employment policies matter. They can redirect economic activities toward employment‐rich sectors, such as health and caring services (D'Alisa et al., 2014). Sharing work by reducing working hours can increase the number of new jobs even if productivity and growth stall (Kallis, Kalush, O'Flynn, Rossiter, & Ashford, 2013). Under certain conditions, shorter working time is linked to lower carbon emissions and other environmental pressures harmful to biodiversity (Knight, Rosa, & Schor, 2013; Shao & Rodríguez‐Labajos, 2016). Biodiversity benefits from reducing working hours are therefore likely (Table S5 in SM2), even if they may depend on complementary policies ensuring that the time liberated from work will not be directed to resource‐intensive consumption (Kallis et al., 2013). Work sharing schemes could be applied in combination with taxation linked to resource use and environmental and biodiversity impacts. Simulations suggest that with a high enough carbon tax, Canada could reduce its carbon emissions by 80% in 2035; while income would contract to the levels of 1976, employment would not decrease if working hours were to be reduced to one fourth of their present level (Victor, 2012).

Another concern is that without economic growth inequality may rise. However, simulations suggest that there is no necessary link between a slowing down of the economy and rising inequality (Jackson & Victor, 2016). Redistributive policies such as high taxes on high‐income brackets, specified ratios for the spread between minimum and maximum salaries, and capital or inheritance taxes can reduce inequality (Piketty, 2014). Without growth in GDP or population, and with an ageing population, societies also face the problem of covering pensions, health care, and education costs. However, the presence of quality health and education systems in middle‐income countries suggests that it is possible to secure good public services at levels of GDP much lower than those of today's rich countries (Gough, 2017).

Relocalizing the economy (Latouche, 2009), namely shortening the distances between production and consumption, is a degrowth principle important for biodiversity conservation, even if local production does not always mean lower environmental impacts (Theurl, Haberl, Erb, & Lindenthal, 2014). Supporting local and regional agroecological management practices that enhance the diversity and services of ecosystems while ensuring food sovereignty could reduce biodiversity pressures from food production (Altieri, 2004; Infante Amate & González de Molina, 2013; Kovács‐Hostyánszki et al., 2017; Table S5 in SM2). While small‐scale farming systems may be less productive in GDP terms, they are employment rich and often provide higher social value for local communities (Jackson, 2011).

Compact urban planning could help limit the physical expansion of cities (Wächter, 2013; Xue, 2014), reducing the ongoing loss and fragmentation of periurban habitats. Periurban croplands—saved from urbanization—could produce food to feed city inhabitants, thus reducing the displacement of agricultural land‐use change to remote biodiverse regions (Marques et al., 2019; Table S5 in SM2). Top‐down national land‐use planning must enforce limits to urban expansion. However, bottom‐up planning schemes are also needed that take into account the regional context, where stakeholders can redesign housing arrangements to solve housing needs while restoring ecosystems (Lietaert, 2010). Finally, labeling based on a product's full biodiversity footprint along international trade routes has the potential to mitigate the impacts of consumption (Lenzen et al., 2012). Together with governmental control of advertisement and the use of public media to provide information on the impacts of products, labeling could contribute to more biodiversity‐friendly consumption (Table S5 in SM2).

Many of these proposed policies have not yet been widely tried nor analyzed, so it is uncertain that they would have the posited effects. The systematic investigation of their prospects constitutes fertile ground for future research.

## 6 FOSTERING THE TRANSITION THROUGH SCENARIO DEVELOPMENT

A range of feasible actions at multiple scales could put humanity on a biodiversity‐friendly pathway while enhancing overall prosperity (Table S5 in SM2). To support this transition, we recommend that in the negotiations of the next CBD COPs and in future assessments of the Intergovernmental Science‐Policy Platform on Biodiversity and Ecosystem Services (IPBES), endorsement of economic growth is replaced by at least a precautionary recognition that it can be problematic for biodiversity. A significant step in this direction has been made in the IPBES Global Assessment Report, by acknowledging the need to move away from the current growth paradigm (IPBES 2019b, p. 19).

At the same time, both CBD and IPBES could act as laboratories where alternative policies are designed, tested, and evaluated through enhanced cooperation between countries, the private sector, and the civil society. Scenario development can play a critical role in this endeavor. Participatory scenario development is suited to overcome the societal addiction to growth as it allows exploring policy options toward a positive vision of a shared future and the commitments necessary to get there (Costanza et al., 2017). Up to now, biodiversity scenarios take growth forecasts as given and search for policy options that can reduce biodiversity loss while the economy grows. Inspired by van den Bergh (2017), we propose here a different approach: first set tight biodiversity targets and then examine how different economic scenarios and conservation policies could accomplish them. This might involve positive, zero, or negative growth. There is no reason to restrict biodiversity policies only to those compatible with positive economic (GDP) growth, as GDP is far from a robust indicator of social welfare (van den Bergh, 2009). Chapter 5 of the IPBES Global Assessment is a good example of the direction this could take (Chan et al., 2019).

**FIGURE 2:**
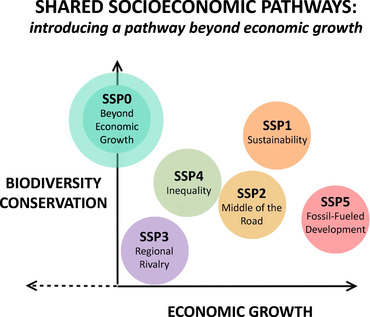
Opening up scenario development for biodiversity conservation. SSPs are descriptions of alternative societal trajectories which are used in scenario development for biodiversity. Here, currently available SSPs (SSP1 to SSP5) are displayed according to their envisaged economic growth rates (in GDP terms) and biodiversity conservation levels (adapted from O'Neill et al., 2017; see this reference for a description of SSPs). Up to now, all SSPs consider positive economic growth rates, and no pathway is included whereby high levels of biodiversity conservation can be achieved with low or negative economic growth. To explore this opportunity space (wider circle in *Y* axis), we propose to add a new SSP called “beyond economic growth” (see Box 2)

The biodiversity scenarios currently under development within IPBES use the shared socioeconomic pathways (SSP) as a basis (Rosa et al., 2017). SSP are descriptions of alternative societal trajectories in demographic, economic, technological, governance, and environmental factors, which serve as inputs to models of climate and other environmental changes (O'Neill et al., 2017). Up to now, all SSPs consider positive economic growth rates, and no pathway is included whereby high levels of social and environmental sustainability can be achieved with low growth (O'Neill et al., 2017). Based on these authors, Figure 2 situates the available SSPs in the bidimensional space “biodiversity conservation” vs. “economic growth” (SSP1 to SSP5). We propose to add a new SSP to examine low, zero, and negative growth pathways compatible with ambitious biodiversity targets and enhanced well‐being (SSP0). This effort could build on already existing scenarios such as the “Great Transition”, which assumes that GDP flattens while well‐being and ecosystem services increase (Great Transition Initiative, 2019; Kubiszewski, Costanza, Anderson, & Sutton, 2017). Box 2 synthesizes our vision for the SSP0.

BOX 2 **A NARRATIVE FOR SSP0. SOURCE: OUR OWN**

The collective awareness of the human embeddedness in the Earth's life network reaches a tipping point. This is triggered by an accumulation of evidence on the social and ecological costs of our development trajectory, as well as by an active inner seeking of genuine well‐being by individuals and countries throughout the world. As a result, humanity initiates a transition to a smaller global economy in material and energetic terms that is able to redistribute wealth and provide enhanced prosperity. The current emphasis on achieving resource efficiency is complemented by the recognition of the need to reduce the overall amount of materials and energy used by the economy. Investment in technology is directed toward liberating time for introspection and learning, not to fuel production and consumption. The demographic transition is accelerated by educational and health investments, curving global population growth. Changing social priorities substitute the consensus around the need for GDP growth for a set of sustainable well‐being indicators, which is adopted by the international community (e.g., in relation to the sustainable development goals). Overall, these changes open up the range of potential biodiversity policies, as these are not constrained anymore to only those compatible with positive GDP growth rates (Figure 2). Scenario development within international biodiversity policies thus explores a broader range of institutional and economic reforms that could accomplish ambitious biodiversity and well‐being targets. By acting as laboratories of new policies, they help to ease the resistance of vested interests against such a transition. New policies include resource caps, resource sanctuaries, limits to large infrastructures, redistributive green taxation, work reduction schemes, agroecological development, compact urban planning and restrictions to advertising. Time liberated from production and consumption of resource intensive products is invested in meditation and self‐awareness. This shift improves overall health levels and deepens the collective awareness of oneness between humans and nature, leading to a positive feedback between human development and ecosystem flourishing.

The use of SSP0 in the IPBES Expert Group on Scenarios and Models could strengthen the discussion on biodiversity policy options. The participatory construction of visions for nature already undertaken by this group echoes calls to replace the pursuit of GDP growth with new well‐being paradigms (Lundquist, Pereira, Alkemade, & den Belder, 2017, p. 21). Yet low, zero, and negative growth pathways have not yet been used in new modeling efforts (Kim et al., 2018), although interest in doing so has been expressed in some regional assessments (IPBES, 2018b).

**FIGURE 3:**
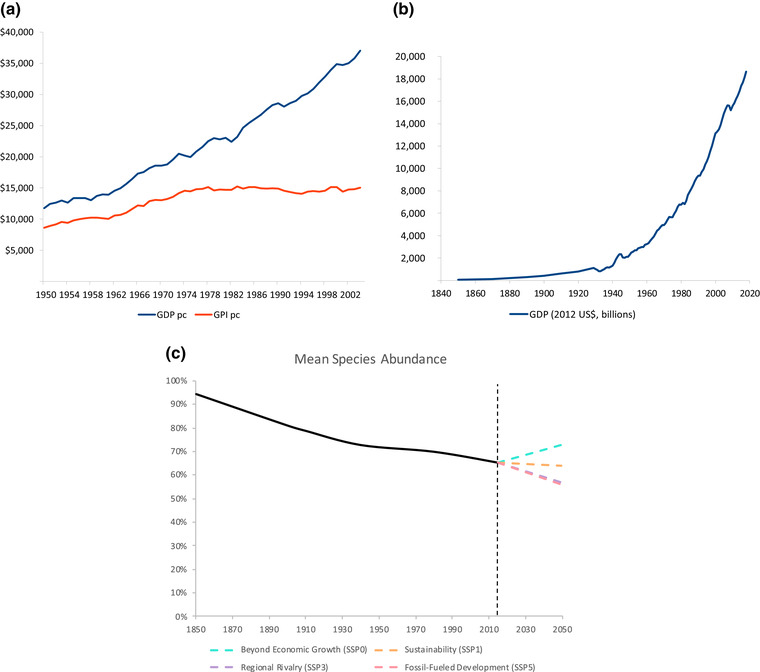
(a) U.S. genuine progress indicator (GPI) and gross domestic product (GDP) per capita, in 2000 U.S.$ (yearly data for 1950–2004). Source: Talberth, Cobb, and Slattery (2007), U.S. Bureau of Economic Analysis (2005), and United Nations (n.d.). (b) U.S. GDP, in billions of 2012 U.S.$ (decennial data for 1850–1920, yearly data for 1929–2018). Source: Barro and Ursúa (2010), United Nations (n.d.), U.S. Bureau of Economic Analysis (2019), and U.S. Census Bureau (1990). (c) Mean species abundance in the U.S. Historical trend (data for years 1850, 1900, 1910, 1940, 1980, and 2015) and projections (2050) for different SSP. Source: Data on historical trend and projections for SSP1, SSP3, and SSP5 were provided by J.P. Hilbers, R. Alkemade, and A.M. Schipper. The value for SSP0 is our speculation (see Section 6 for details on SSP0). See SM3 for details on figures' sources and methods.

In this endeavor, the use of an integrated set of metrics composed of economic measures, social indicators, biophysical indicators (including biodiversity), subjective measures of well‐being, and composite measures of several indicators (Costanza et al., 2016, 2014; O'Neill, 2012; O'Neill, Fanning, Lamb, & Steinberger, 2018; Czech et al., 2005) would encourage a better understanding of the relationships between economic activity, social well‐being, and biodiversity. The fear that achieving ambitious conservation targets is likely to diminish GDP could be calmed by visualizing stable or even positive trends in more robust measures of well‐being such as the genuine progress indicator (GPI) (Kubiszewski et al., 2013; Talberth & Weisdorf 2017; see Figure 3; Box 3). In addition, it would be important to account for ecosystems’ positive contributions to well‐being in new metrics where sustainable and equitable prosperity is the explicit goal (Costanza et al., 2016). The global transdisciplinary research effort on nature's contributions to people (Díaz et al., 2018) offers a valuable resource for advancing this work. While changing modeling paradigms is not changing policy, adding an SSP0 to current scenario analyses could contribute to overcome the growth dependency of countries and help them shift their political economic priorities toward better biodiversity and well‐being policies.

BOX 3 **A SHIFT IN BIODIVERSITY SCENARIO DEVELOPMENT IN POLICY FORU MS. SOURCE: OUR OWN, WITH DATA MENTIONED IN THE TEXT**

Whereas U.S. GDP per capita experienced an almost continuous upward trend since 1950, GPI per capita increased steadily until about 1978 and flattened out (Talberth, Cobb, & Slattery, 2007; Kubiszewski et al., 2013; Figure 3a). Economic growth seems to bring about an improvement in social well‐being but only up to a certain threshold. Instead, there is no “threshold” for biodiversity degradation. The number of threatened and endangered species has increased sharply since the 1970s (Czech et al., 2005) and the mean species abundance has continuously declined over the period 1850–2015 alongside a growing economy (Figures 3b and 3c). The evidence presented in this paper suggests a strong connection between endless growth and biodiversity loss. Moreover, the growing economy is reducing ecosystem's contribution to well‐being (Costanza et al., 2016; Kubiszewski et al., 2013). Projections for MSA in 2050 using currently available SSPs show that, at best, we could keep biodiversity degradation at levels similar to those of 2015 (SSP1, Figure 3c). SSP1 is a green growth scenario that relies either on negative emissions technologies—unproven and dangerous at scale—or unfeasible decarbonisation rates (Doelman et al., 2018; Hickel & Kallis, 2019). An alternative option to SSP1 is to first set biodiversity targets and then examine which combinations of economic growth and conservation policies could accomplish them. If, for example, by 2050 we were to recover MSA to 1940 levels (73%; SSP0 in Figure 3c), what GDP growth rate would be consistent with such target? What could be the combined contribution of conservation policies like resource caps, land‐use regulations, and agroecological development schemes? How would GPI and other measures of well‐being react to redistributive green taxation, work‐sharing programs, and a recovery of ecosystems?

## 7 CONCLUSION

Economic growth and biodiversity loss are linked via a set of mechanisms triggered by increased resource use. While absolute decoupling remains a theoretical possibility, it has not occurred so far and seems unlikely to occur in the near future in the absence of major transformations in the economic system. By contrast, global biodiversity and sustainability policies generally advocate economic growth and have ambiguous positions regarding its effects on biodiversity. This reflects the widespread assumption that growth is needed to secure prosperity, despite increasing evidence that, under certain conditions, high levels of social well‐being may be achievable without—or beyond—growth. Scenario development can play a critical role in shifting away from the current development model, whereby positive visions of a shared future are collectively designed. In particular, we propose that a new SSP is introduced that examines low, zero, or negative growth pathways compatible with ambitious biodiversity and well‐being targets. Using this SSP0 within IPBES—which will advise the CBD during the adoption and implementation of a post‐2020 framework for biodiversity—has the potential to open up the range of policy options beyond mere projections of the status quo. The discussion on crucial aspects of this framework—new targets and indicators, mainstreaming of biodiversity across all economic sectors and transformative change—can benefit from both the evidence and the alternative scenarios presented in this paper.

## Supporting information


**Table S1**. Criteria used to classify policy documents for core analytical categories *a*, *b* and *c*.
**Table S2**. Search words used to identify reasons behind the policy documents’ views on economic growth and its relationship to environmental problems. After each reason, in brackets, the sign indicates whether that reason is given for advocating (+) or cautioning against (‐) economic growth. For some search words, the character of the causal link between economic growth and that particular variable is indicated in brackets.
**Table S3**. Search words used to identify the policy documents’ view on the relationship between economic growth and biodiversity based on drivers of biodiversity loss. For some search words, the specific meaning that was searched for is indicated in brackets.
**Table S4**. Results of the content analysis of key international agreements and declarations on biodiversity and the environment, regarding their positions on economic growth, biodiversity impacts and decoupling. NA = Not assessed.
**Table S5**. Biodiversity beyond economic growth: seven policy proposals. Source: our own interpretation of the references mentioned in the table.
Figure 3a: Based on Talberth et al. (2007).
Figure 3b: Yearly real GDP data from 1929 to 2018 (in chained 2012 US$) were obtained from the U.S. Bureau of Economic Analysis (2019).
Figure 3c: Mean Species Abundance (MSA) in the US.

## ENDNOTES

1
*Report of the World Commission on Environment and Development. Our Common Future*. United Nations, 1987 (articles 24 and 72, chapter 3 “The Role of the International Economy”).

2
*The Rio Declaration on Environment and Development*. United Nations Conference on Environment and Development, 1992 (principle 12).

3
*Towards a Green Economy: Pathways to Sustainable Development and Poverty Eradication*. Nairobi: United Nations Environment Program, 2011 (p. 16). This document is not included in our policy review (see SM1).

4
*Resolution adopted by the General Assembly on 27 July 2012*. United Nations, A/RES/66/288 (p. 2).

5
*Resolution adopted by the General Assembly on 25 September 2015*. United Nations, A/RES/70/1 (goal 8).

6
*Cancun Declaration on Mainstreaming the Conservation and Sustainable Use of Biodiversity for Well‐Being*. Cancun, Mexico: 13th meeting of the Conference of the Parties to the Convention on Biological Diversity, 2016 (declaration 4 and commitment 7).

7
*Global Biodiversity Outlook 4*. Montréal: Secretariat of the Convention on Biological Diversity, 2014 (pp. 12 and 45).

8
*Decision adopted by the Conference of the Parties to the Convention on Biological Diversity at its Tenth Meeting*. Convention on Biological Diversity, 2010, UNEP/CBD/COP/DEC/X/2 (target 4).
