# Supplementary material for: Biodiversity policy beyond economic growth
Source: Conserv Lett. 2020 Apr 13;13(4):e12713. doi: 10.1111/conl.12713 (PMC7507775; doi:10.1111/conl.12713)
Supplement: Supplementary file 1 — Table S1. Criteria used to classify policy documents for core analytical categories a, b and c. Table S2. Search words used to identify reasons behind the policy documents’ views on economic growth and its relationship to environmental problems. After each reason, in brackets, the sign indicates whether that reason is given for advocating (+) or cautioning against (‐) economic growth. For some search words, the character of the causal link between economic growth and that particular variable is indicated in brackets. Table S3. Search words used to identify the policy documents’ view on the relationship between economic growth and biodiversity based on drivers of biodiversity loss. For some search words, the specific meaning that was searched for is indicated in brackets. Table S4. Results of the content analysis of key international agreements and declarations on biodiversity and the environment, regarding their positions on economic growth, biodiversity impacts and decoupling. NA = Not assessed. Table S5. Biodiversity beyond economic growth: seven policy proposals. Source: our own interpretation of the references mentioned in the table. Figure 3a: Based on Talberth et al. (2007). Figure 3b: Yearly real GDP data from 1929 to 2018 (in chained 2012 US$) were obtained from the U.S. Bureau of Economic Analysis (2019). Figure 3c: Mean Species Abundance (MSA) in the US. [file CONL-13-e12713-s001.docx]

Supplementary Material for: Otero I, Farrell KN, Pueyo S, et al. Biodiversity policy beyond economic growth. *Conservation Letters*. 2020;e12713. https://doi.org/10.1111/conl.12713

**Supplementary Material 1. Internal contradictions on economic growth and biodiversity conservation in key international declarations and agreements on biodiversity and the environment**

1. Rationale of the analysis of policy documents

A systematic document review procedure was used to highlight internal contradictions in the positions presented on economic growth and biodiversity conservation in key international declarations and agreements on biodiversity and the environment. A systematic review of the documents was conducted, according to clearly formulated questions, and using systematic and explicit criteria for selecting and appraising relevant content (Petticrew & Roberts, 2006). The selection of documents for this review was based on their relevance to global level biodiversity policy and governance, with a total of 28 key international declarations and agreements on biodiversity and the environment selected for review. Documents were analysed using three core analytical categories: position on economic growth and the environment, position on economic growth and biodiversity, and position on decoupling (Table S1). Positions were determined by searching for the presence of selected keywords (detailed in Tables S2, S3) and by evaluating the semantic context in which these words were embedded. Based on this review, the general narrative of each document was assessed with regards to the three core analytical categories. These positions were then used to identify the presence or absence of internal contradictions with respect to the documents’ advocacy of economic growth and their concerns regarding how growth is linked to the drivers of biodiversity loss.

2. Document selection

To ensure breadth and depth of analysis, key documents concerned specifically with biodiversity (21) and key documents concerned with the environment and sustainability (7) were surveyed. The selected documents cover the period 1972-2016, which extends from the first Earth summit, held in Stockholm, to the second to last Conference of the Parties (COP) to the Convention on Biological Diversity (CBD), held in Cancun^[[1]](#footnote-1)^.

21 key documents concerned specifically with biodiversity were selected, based on general relevance, status as precedent, and status as most current. They included: i) two globally relevant general documents: the original CBD from 1992 and the most recent Global Biodiversity Outlook Report from 2014; ii) twelve official COP Final Reports (COP 1 to COP 12) and the opening statement by the CBD’s Executive Secretary to the COP 13^[[2]](#footnote-2)^; iii) all three official protocols related to the CBD, i.e. the Cartagena (2000), Nagoya (2011), and Kuala-Lumpur (2011) Protocols; iv) the 2010 COP 10 document outlining the Strategic Plan 2011-2020 and its associated Aichi Targets; and v) the 2014 Gangwon Declaration (COP 12) and the 2016 Cancun Declaration (COP 13).

Seven key global documents concerned with the environment and sustainability, in general, were also selected, based on the universality of their recognition. They included: i) the four major Intergovernmental Agreements concerning human impacts upon the Earth’s environment, starting with the Stockholm Agreement of 1972, followed by the Rio Agreement of 1992, the Johannesburg Agreement of 2002 and the Rio+20 Agreement of 2012; and ii) another three major documents concerning the state of the world’s environment and the actions required in order to improve the situation, starting with the Brundtland Report of 1987, followed by the Millennium Ecosystem Assessment of 2005, and the Sustainable Development Goals Resolution of 2015. All these documents had chapters or passages devoted to biodiversity loss and conservation.

3. Analytical categories and associated criteria

Three logically dependant core analytical categories were employed: a) view on economic growth and its relationship to environmental problems; b) view on the relationship between economic growth and biodiversity; and c) view on decoupling economic growth from biodiversity loss. Broad search categories were used in order to allow for comparison across the range of selected documents, which were produced over a period of 50 years, of varied lengths, and authored by different groups of authors.

For the core categories a) and b), each surveyed document was assigned one of the following labels: problematic, unproblematic or ambiguous. For the core category c), each surveyed document was assigned one of the following labels: challenging, unchallenging or ambiguous. The criteria used to assign these labels are detailed in Table S1. Analytical categories and criteria were first developed by a team of three authors, which later analysed one third of the selected documents each. Results were tested, reviewed and cross-checked in team meetings. In combination with a final review by one of them (the first author of the paper), this ensured analytical consistency across documents.

4. Search procedure

In order to label the selected documents according to the criteria detailed in Table S1, each document was reviewed using search words. First, general words such as ‘growth’ (category *a*) and ‘biodiversity’ (category *b*) were used. Then, specific search words were used for category *a* (shown in Table S2) and category *b* (shown in Table S3). When a search word was found, the relevance of its semantic context was checked. For instance, the physical growth of plants was discussed in many of the searched documents; however the meaning with which the word was used in these particular instances was not deemed relevant for the analytical aim of the review and so the match was disregarded.

Search words for category *a* (Table S2) were aimed at capturing the implicit or explicit reasons underlying the documents’ stance on economic growth and on the relationship between economic growth and environmental problems. These reasons were identified in a preliminary content analysis of a subsample of documents, where three archetypical lines of argumentation were found to be common: i) economic growth alleviates poverty; ii) economic growth fosters development; and iii) economic growth threatens to exceed the Earth’s biophysical limits. A particular reason was assigned if and only if both a search word belonging to that reason was found and it occurred in a semantic context deemed relevant to the search. A document could be assigned none, one, two or three reasons. Assessment of category *a* was thus based on the reasons given in the document for advocating or cautioning against economic growth.

Search words for category *b* (Table S3) were aimed at capturing the drivers of biodiversity loss identified within the documents, as well as the documents’ general stance on the relationship between economic growth and biodiversity loss. Three categories of drivers of biodiversity loss were created based on the evidence presented in the main text of this article: i) material and energetic expansion, ii) land-use change, and iii) climate change, all driven by economic growth (see main text). A particular category of drivers was assigned if and only if a search word belonging to that driver was found and it occurred in a semantic context deemed relevant to the search. Besides the specified search words, quasi-synonyms were also used as search words to account for the different ways in which the drivers of biodiversity loss were referred to in the documents. For instance, ‘habitat loss’ and ‘deforestation’ were searched for as they are quasi-synonyms of ‘habitat destruction’ and ‘forest loss’, respectively. When a reference to a driver was found that had (almost) the same meaning as one of the search words specified in Table S3, the corresponding category of drivers was assigned. For instance, when ‘livestock production’ was found as a driver of biodiversity loss identified by the document, the category ‘material and energetic expansion’ was assigned because of similarity with search word ‘meat’. Assessment of category *b* was based on reference to these drivers, as well as on general statements regarding the effects of economic growth on biodiversity.

The views on decoupling economic growth from biodiversity loss (category *c*) within the selected documents were searched for in statements found within the semantic contexts of the search words, while conducting the search explained above. Specific search words for decoupling were also used, including ‘decoupl’ and a small set of additional words such as ‘efficien’ (for efficiency) and ‘demater/de-mater’ (for dematerialization). These additional search words were identified through review of the Brundtland Report from 1987, which was written before the term ‘decoupling’ came into wide use. In the Brundtland Report, these search words occurred in semantic contexts expressing the document’s vision concerning prospects for reducing the impacts of resource use while continuing economic growth, i.e. what it is currently understood by ‘decoupling’.

All search words used in the search were employed in a time-sensitive manner, i.e. by using also variations of the word more commonly used at the time when the document was written. For instance, for those documents written before ‘biodiversity’ came into common use, ‘biological diversity’ and ‘biosphere’ were used as substitutes. Similarly, ‘climatic change’ was used as a substitute of ‘climate change’ in those documents in which the former was the preferred form. A priori, each search word was searched for throughout the entirety of each document, in order to support the development of a complete picture of how the word was used, by and large, in relation to the analysis, within the document. However, a saturation criterion was used in search words for categories *a* and *b*. According to this, when search words started yielding redundant information within one particular category, and the general narrative of the text was clear, the search for that category was abandoned. For instance, in the Brundtland report, the advocacy of economic growth as a means to foster (sustainable) development became apparent before all search words hits for the category ‘development’ in the document had been checked. Similarly, the three categories of drivers of biodiversity loss were straightforwardly identified in the Millennium Ecosystem Assessment when searching for ‘biodiversity’, making it unnecessary to use all the specific search words because the objective of the search was fulfilled. In cases where a particular category is not assigned, all the search words for that category have been exhausted.

5. Content analysis and assessment of contradictions

Based on review of the semantic context within which search words were embedded, the general narrative of the text was evaluated, with regards to: i) whether or not economic growth and decoupling of economic growth from biodiversity loss were mentioned (yes or no); and ii) what the general orientation of the document was, with regard to the three core analytical categories (Table S1). Documents were considered to mention economic growth when they did so in an explicit way and also when they used other words to convey the same meaning, i.e. expansion of economic production and consumption (e.g. ‘economic development’). Results are presented in Table S4.

Based on these results, contradictions in the documents’ positions concerning economic growth and biodiversity conservation were assessed (also shown in Table S4). Documents were classified as not exhibiting internal contradictions if i) economic growth was viewed as unproblematic for the environment (category *a*), and ii) none of the expected biodiversity loss drivers were identified; or if i) economic growth was viewed as problematic for environment (category *a*) and ii) one or more of the expected biodiversity loss drivers was identified. Documents were classified as containing internal contradictions if i) economic growth was viewed as unproblematic for the environment, or the position on this was ambiguous (category *a*), and ii) one or more of the biodiversity loss drivers were identified in the document.

**Table S1.**

Criteria used to classify policy documents for core analytical categories *a*, *b* and *c*.

|  | **a. View on economic growth and its relationship to environmental problems** | **b. View on the relationship between economic growth and biodiversity** |  | **c. View on decoupling economic growth from biodiversity loss** |
| --- | --- | --- | --- | --- |
| Problematic if | growth is presumed to be correlated with environmental problems | growth is explicitly presumed to have either a negative, or potentially negative, impact on biodiversity | Challenging if | decoupling economic growth from biodiversity loss is explicitly presumed to be complicated, difficult, or potentially impossible |
|  |  |  |  |  |
| Unproblematic if | growth is presumed either to not be correlated with environmental problems or to be a solution to them (e.g. environmental Kuznets Curve) | growth is presumed to have either no impact or a positive impact on biodiversity | Unchallenging if | decoupling economic growth from biodiversity loss is explicitly presumed to be easy and automatic |
| Ambiguous if | the position is either internally contradictory, sometimes seen as problematic sometimes not, or too vague to be determined | the position is either internally contradictory, sometimes seen as problematic sometimes not, or too vague to be determined | Ambiguous if | the position is either internally contradictory, sometimes seen as problematic sometimes not, or too vague to be determined |

**Table S2.**

Search words used to identify reasons behind the policy documents’ views on economic growth and its relationship to environmental problems. After each reason, in brackets, the sign indicates whether that reason is given for advocating (+) or cautioning against (-) economic growth. For some search words, the character of the causal link between economic growth and that particular variable is indicated in brackets.

| Reason | **Poverty (+)** | **Development (+)** | **Limits (-)** |
| --- | --- | --- | --- |
| Search words | (reduce) poverty  (reduce) hunger  (reduce) child mortality  (improve) housing  (improve) sanitation  (improve) education  (increase) literacy  (increase) life expectancy | develop  economic grow  (increase) GDP  (improve) quality of life  (increase) life expectancy  (increase) income  (improve) health | limit/s  footprint  food  carbon  phosphor  nitrogen  land  water |

**Table S3.**

Search words used to identify the policy documents’ view on the relationship between economic growth and biodiversity based on drivers of biodiversity loss. For some search words, the specific meaning that was searched for is indicated in brackets.

| Drivers’ category | **Material / Energetic expansion** | **Land-use change** | **Climate change** |
| --- | --- | --- | --- |
| Search words | material  energy  consumption  production  extraction  contamination  meat  fossil fuel  waste  population  species (trade)  hunting  poaching  pollution | land-use change  habitat (destruction)  urban  infrastructure  forest conversion  forest loss  plantation  agriculture  mining | climate change  extreme weather  drought  flood  temperature  rainfall  variability  erosion  landslides  ocean acidification  rising sea-level  ice-melt  mitigation |

**Table S4.**

Results of the content analysis of key international agreements and declarations on biodiversity and the environment, regarding their positions on economic growth, biodiversity impacts and decoupling. NA = Not assessed.

|  | **Document** | **a. View on economic growth and its relationship to environmental problems** | | | **b. View on the relationship between economic growth and biodiversity** | | **c. View on decoupling economic growth from biodiversity loss** | | **Contradictions** |
| --- | --- | --- | --- | --- | --- | --- | --- | --- | --- |
|  |  | **Is economic growth mentioned?** | **View** | **Reasons given** | **View** | **Biodiversity loss drivers identified** | **Is decoupling mentioned?** | **View** |  |
| General documents on the environment and sustainability | Declaration UN Conference on the Human Environment Stockholm (1972) | Yes | Ambiguous | Development; Poverty; Limits | Problematic | Material/energy; Land-use change | Yes | Challenging | Yes |
|  | UN Report of the World Commission on Environment and Development (1987) (Brundtland Report) | Yes | Ambiguous | Development; Poverty; Limits | Ambiguous | Material/energy; Land-use change | Yes | Challenging | Yes |
|  | Declaration UN Conference on Environment and Development Rio de Janeiro (1992) | Yes | Unproblematic | Development; Poverty; Limits | Ambiguous | Material/energy; Land-use change; Climate change | No | NA | Yes |
|  | Declaration UN World Summit on Sustainable Development Johannesburg (2002) | Yes | Unproblematic | Development; Poverty | Unproblematic | Material/energy; Climate change | Yes | Unchallenging | Yes |
|  | Millennium Ecosystem Assessment (2005) | Yes | Ambiguous | Limits | Ambiguous | Material/energy; Land-use change; Climate change | Yes | Challenging | Yes |
|  | Declaration UN Conference on Sustainable Development Rio de Janeiro (2012) (Rio + 20) | Yes | Unproblematic | Development; Poverty | Problematic | Material/energy; Land-use change; Climate change | No | NA | Yes |
|  | UN Sustainable Development Goals (2015) | Yes | Unproblematic | Development | Ambiguous | Material/energy; Land-use change; Climate change | Yes | Unchallenging | Yes |
| Biodiversity specific documents | Convention on Biological Diversity (1992) | Yes | Ambiguous | Development; Poverty | Ambiguous | Land-use change | No | NA | Yes |
|  | Report CBD COP 1 (1994) | Yes | Ambiguous | Development; Limits | Ambiguous | Land-use change; Climate change | No | NA | Yes |
|  | Report CBD COP 2 (1995) | No | NA | NA | Problematic | Material/energy; Land-use change | No | NA | NA |
|  | Report CBD COP 3 (1996) | Yes | Ambiguous | Development | Ambiguous | Material/energy; Land-use change | Yes | Challenging | Yes |
|  | Report CBD COP 4 (1998) | Yes | Unproblematic | Development; Poverty | Ambiguous | Material/energy; Land-use change; Climate change | Yes | Challenging | Yes |
|  | Report CBD COP 5 (2000) | Yes | Ambiguous | Development; Poverty; Limits | Ambiguous | Material/energy; Land-use change; Climate change | No | NA | Yes |
|  | Cartagena Protocol on Biosafety to the CBD (2000) | Yes | Unproblematic | Development | Unproblematic | Land-use change | No | NA | Yes |
|  | Report CBD COP 6 (2002) | Yes | Ambiguous | Limits | Problematic | Material/energy | No | NA | Yes |
|  | Report CBD COP 7 (2004) | Yes | Unproblematic | Poverty | Unproblematic | Material/energy; Land-use change; Climate change | No | NA | Yes |
|  | Report CBD COP 8 (2006) | Yes | Ambiguous | Development; Poverty; Limits | Ambiguous | Material/energy; Land-use change; Climate change | Yes | Challenging | Yes |
|  | Report CBD COP 9 (2008) | Yes | Ambiguous | Development; Poverty | Ambiguous | Material/energy; Land-use change; Climate change | No | NA | Yes |
|  | Report CBD COP 10 (2010) | Yes | Ambiguous | Development; Poverty | Ambiguous | Material/energy; Land-use change; Climate change | Yes | Ambiguous | Yes |
|  | Strategic Plan 2011-2020 and Aichi Targets CBD COP 10 (2010) | Yes | Unproblematic | Development; Poverty | Unproblematic | Material/energy; Land-use change; Climate change | No | NA | Yes |
|  | Nagoya - Kuala Lumpur Supplementary Protocol to Cartagena Protocol (2011) | No | NA | NA | Ambiguous | None | No | NA | NA |
|  | Nagoya Protocol on Access to Genetic Resources to the CBD (2011) | Yes | Unproblematic | Development; Poverty | Unproblematic | Material/Energy; Climate change | No | NA | Yes |
|  | Report CBD COP 11 (2012) | Yes | Problematic | Development; Poverty; Limits | Problematic | Material/energy; Land-use change; Climate change | Yes | Ambiguous | No |
|  | Report CBD COP 12 (2014) | Yes | Ambiguous | Development; Poverty | Ambiguous | Material/energy; Land-use change; Climate change | No | NA | Yes |
|  | Gangwon Declaration CBD COP 12 (2014) | No | NA | NA | NA | None | No | NA | NA |
|  | Global Biodiversity Outlook 4 (2014) | Yes | Problematic | Limits | Problematic | Material/energy; Land-use change; Climate change | Yes | Challenging | No |
|  | Opening statement to CBD COP 13 (2016) | No | NA | NA | NA | None | No | NA | NA |
|  | Cancun Declaration CBD COP 13 (2016) | Yes | Problematic | Development; Poverty; Limits | Ambiguous | Material/energy; Land-use change; Climate change | Yes | Unchallenging | No |

**Supplementary Material 2. Biodiversity policies beyond economic growth**

**Table S5.** Biodiversity beyond economic growth: seven policy proposals. Source: our own interpretation of the references mentioned in the table.

| **Policy proposal** | **Direction and scale of implementation** | | **Hypothesized effect on biodiversity pressures** |
| --- | --- | --- | --- |
|  | **Top-down: sub-national, national, and international action** | **Bottom-up: indigenous and local communities (ILC), NGOs, researchers and companies** |  |
| 1. Caps to the resource- and land-content of internationally traded products (Alcott, 2010). | Establishment of absolute caps on the amount of product-embodied resources and land that a country can import or export. | Researchers provide the best available knowledge on international resource flows while monitoring the effects of implemented caps. | Less international trade reduces resource extraction and the expansion of invasive species. |
| 2. Resource sanctuaries and limits to extractive industries in biodiverse areas (Videira et al., 2014). | Set clearer limitations and remove subsidies to harmful extractive industries. Possible moratoria on resource extraction in highly sensitive regions (sanctuaries). Taxation of products should reflect their impact to promote a less resource intensive production. | ILC mobilize for the conservation of their livelihood base and biocultural diversity, supported by environmental justice NGOs. Researchers provide knowledge to identify and implement sanctuaries at different scales, while offering pragmatic institutional and livelihood alternatives to economic growth. | Reduced extraction of material and energy slows down habitat loss and fragmentation. |
| 3. Limits to large infrastructures (Videira et al., 2014; Ibisch et al., 2016). | Carefully (re-)examine the need of new large infrastructures (airports, dams, motorways) and their impact on sensitive ecosystems and human communities. | Community-based conservation can be optimized through collaboration between ILC affected by infrastructure expansion, social and environmental NGOs and researchers. The latter contribute with knowledge and tools to identify which infrastructures could be limited, where, and why. | Protecting the remaining tracts of road free areas helps halting the rapid loss of key biodiversity refugia and protect at-risk cultures. |
| 4. Work reduction and sharing (Kallis et al., 2013; Knight et al., 2013; Shao & Rodríguez-Labajos, 2016). | Legislation to reduce the working week and to support companies that facilitate work sharing. | Companies implement work sharing schemes in collaboration with trade unions. Reduction of working hours offers employment for more people. | Shorter working time is associated with smaller environmental pressures, entailing lower biodiversity impacts. |
| 5. Agro-ecology and food sovereignty (Altieri, 2004; Infante Amate & González de Molina, 2013; Kovács-Hostyánszki et al., 2017). | Governmental support to sustainable farming systems, and local and organic foods via regulations and subsidies. Taxation systems should be adjusted to reflect resource and waste flows associated with food production. | Initiatives to promote slow, local and organic foods that shorten production chains. Scientists can help by developing biodiversity and sustainability criteria linked to available evidence, as well as monitoring programs. | Pressures on land from agriculture and livestock production are reduced. Intensive organic production in agro-ecosystems promotes intraspecific, interspecific and landscape diversity. |
| 6. Compact urban planning and house sharing (Lietaert, 2010; Wächter, 2013; Xue, 2014). | Promote efficient land use through supporting collective housing arrangements, rent controls in the face of gentrification, caps on the amount of land made available for urbanization, and limits to peri-urban expansion. | Initiatives by neighbourhoods, urban- and land-planners and architects can (re)design housing arrangements. Private, cooperative or public-private arrangements can develop integrated solutions to address housing needs while preserving and monitoring (semi-)natural ecosystems in and around cities. | Lower land consumption for housing and related infrastructures reduces habitat loss and fragmentation, while promoting nature restoration in urban areas. Reduced pressure from urbanization on peri-urban farmland reduces the displacement of agricultural land-use change to biodiverse regions. |
| 7. Enhance information on biodiversity impacts of products and control advertising (Lenzen et al., 2012; Videira et al., 2014). | Governments could tax advertisements of products entailing overexploitation of species and land, while promoting the use of public media to provide information on the biodiversity impacts of goods and services. | Cooperation between companies and consumers can enhance the awareness on the biodiversity impacts of products through improved labelling and information campaigns. Citizen-science initiatives can trace the impacts of products to support labelling, which can be combined with education programmes to foster responsible consumption. | More responsible consumption reduces the demand for products that put high pressure on biodiversity. |

**Supplementary Material 3. Figures’ sources and methods**

Figure 3a: Based on Talberth et al. (2007). Data on GPI per capita come from this source. Data on GDP per capita result from combining the GDP data in U.S. Bureau of Economic Analysis (2005) with the population data in United Nations (n.d.).

Figure 3b: Yearly real GDP data from 1929 to 2018 (in chained 2012 US$) were obtained from the U.S. Bureau of Economic Analysis (2019). Decennial GDP data from 1850 to 1920 were obtained based on the historical GDP pc series compiled by Barro and Ursúa (2010). This series consists of percentages of 2006 GDP pc, using PPP-adjusted value in 2000 international dollars, and gives annual data from 1790 to 2009. Combining this series with the series of U.S. population which runs from 1948 to 2016 (United Nations, n.d.), a series of GDP in arbitrary units was obtained for 1948-2009. Given the high correlation with the mentioned BEA GDP data for this period (r^2^=0.9998), a coefficient to estimate the latter from the former was obtained by linear regression through the origin. Another input was the time series of U.S. population at intervals of 10 years from 1790 to 1990 (U.S. Census Bureau, 1990). The population values from 1850 to 1920 were multiplied by the values of GDP pc for the corresponding years in Barro and Ursúa (2010) and by the coefficient that we fitted, thus obtaining estimated real GDP in 2012 US$ for this earlier period too.

Figure 3c: Mean Species Abundance (MSA) in the US. The figure shows the historical trend (data for years 1850, 1900, 1910, 1940, 1980 and 2015) and projected trajectories for 2050. MSA is a measure of local biodiversity intactness. Values given are based on land use and climate change effects. Projections refer to three available Shared Socioeconomic Pathways (SSP) coupled with three Representative Concentration Pathways (RPC). SSP1xRCP2.6 is characterized by moderate growth in consumption, limited land-use change due to regulation, substantial improvements in agricultural productivity allowing for reforestation, and low greenhouse gas emissions consistent with the 2ºC increase target. SSP3xRCP6.0 is characterized by low or no economic growth, high population growth, limited regulation of land-use change leading to continued deforestation, and intermediate greenhouse gas emissions. SSP5xRCP8.5 is characterized by strong economic growth, a consumption-oriented and energy-intensive society, highly intensive agricultural practices leading to a decline in deforestation, and high greenhouse gas emissions (Data provided by J.P. Hilbers, R. Alkemade and A.M. Schipper; see Kim et al. (2018) for the methodology, and Schipper et al. (2020) for details on MSA). The projected value for SSP0 is our speculation. SSP0 is characterized by a shift in political priorities from economic growth to sustainable social wellbeing, and low greenhouse gas emissions consistent with the 1.5°C increase target. It includes strong limits to land-use change and measures leading to large-scale ecosystem recovery, including land abandonment (see Section 6 for details).

**References**

Alcott, B. (2010). Impact caps: Why population, affluence and technology strategies should be abandoned. *Journal of Cleaner Production*, *18*(6), 552–560. https://doi.org/10.1016/j.jclepro.2009.08.001

Altieri, M. A. (2004). Linking ecologists and traditional farmers in the search for sustainable agriculture. *Frontiers in Ecology and the Environment*, *2*(1), 35–42. https://doi.org/10.1890/1540-9295(2004)002[0035:LEATFI]2.0.CO;2

Barro, R. J., & Ursúa, J. F. (2010). *Barro-Ursúa macroeconomic data*. http://scholar.harvard.edu/barro/publications/barro-ursua-macroeconomic-data (accessed 08/01/2013)

Ibisch, P. L., Hoffmann, M. T., Kreft, S., Pe’er, G., Kati, V., Biber-Freudenberger, L., … Selva, N. (2016). A global map of roadless areas and their conservation status. *Science*, *354*(6318), 1423–1427. https://doi.org/10.1126/science.aaf7166

Infante Amate, J., & González de Molina, M. (2013). ‘Sustainable de-growth’ in agriculture and food: An agro-ecological perspective on Spain’s agri-food system (year 2000). *Journal of Cleaner Production*, *38*, 27–35. https://doi.org/10.1016/j.jclepro.2011.03.018

Kallis, G., Kalush, M., O’Flynn, H., Rossiter, J., & Ashford, N. (2013). ‘Friday off’: Reducing working hours in Europe. *Sustainability*, *5*, 1545–1567.

Kim, H., Rosa, I. M. D., Alkemade, R., Leadley, P., Hurtt, G., Popp, A., … Pereira, H. M. (2018). A protocol for an intercomparison of biodiversity and ecosystem services models using harmonized land-use and climate scenarios. *Geoscientific Model Development*, *11*(11), 4537–4562. https://doi.org/10.5194/gmd-11-4537-2018

Knight, K. W., Rosa, E. A., & Schor, J. B. (2013). Could working less reduce pressures on the environment? A cross-national panel analysis of OECD countries, 1970–2007. *Global Environmental Change*, *23*(4), 691–700. https://doi.org/10.1016/j.gloenvcha.2013.02.017

Kovács-Hostyánszki, A., Espíndola, A., Vanbergen, A. J., Settele, J., Kremen, C., & Dicks, L. V. (2017). Ecological intensification to mitigate impacts of conventional intensive land use on pollinators and pollination. *Ecology Letters*, *20*(5), 673–689. https://doi.org/10.1111/ele.12762

Lenzen, M., Moran, D., Kanemoto, K., Foran, B., Lobefaro, L., & Geschke, A. (2012). International trade drives biodiversity threats in developing nations. *Nature*, *486*(7401), 109–112. https://doi.org/10.1038/nature11145

Lietaert, M. (2010). Cohousing’s relevance to degrowth theories. *Journal of Cleaner Production*, *18*(6), 576–580. https://doi.org/10.1016/j.jclepro.2009.11.016

Petticrew, M., & Roberts, H. (2006). *Systematic reviews in the social sciences: A practical guide*. Oxford, MA: Blackwell Publications

Schipper, A. M., Hilbers, J. P., Meijer, J. R., Antão, L. H., Benítez‐López, A., Jonge, M. M. J. de, … Huijbregts, M. A. J. (2020). Projecting terrestrial biodiversity intactness with GLOBIO 4. *Global Change Biology*, *26*(2), 760-771. https://doi.org/10.1111/gcb.14848

Shao, Q., & Rodríguez-Labajos, B. (2016). Does decreasing working time reduce environmental pressures? New evidence based on dynamic panel approach. *Journal of Cleaner Production*, *125*, 227–235. https://doi.org/10.1016/j.jclepro.2016.03.037

Talberth, J., Cobb, C., & Slattery, N. (2007). *The Genuine Progress Indicator 2006. A tool for sustainable development*. Oakland, CA: Redefining progress.

United Nations (n.d.). *UNdata*. http://data.un.org/Data.aspx?d=POP&f=tableCode%3a1 (accessed 04/10/2019)

U.S. Bureau of Economic Analysis (2005). *National accounts (NIPA) March 30, 2005*. https://apps.bea.gov/histdata/fileStructDisplay.cfm?HMI=7&DY=2004&DQ=Q4&DV=3.%20Final&dNRD=March-30-2005 (accessed 04/11/2019)

U.S. Bureau of Economic Analysis (2019). *National accounts (NIPA) October 31, 2019*. https://apps.bea.gov/histdata/fileStructDisplay.cfm?HMI=7&DY=2019&DQ=Q3&DV=Advance&dNRD=October-31-2019 (accessed 05/11/2019)

U.S. Census Bureau (1990). *Selected historical decennial census population and housing counts. Population, housing units, area measurements, and density: 1790 to 1990*. https://www.census.gov/population/www/censusdata/hiscendata.html (accessed 24/11/2019)

Videira, N., Schneider, F., Sekulova, F., & Kallis, G. (2014). Improving understanding on degrowth pathways: An exploratory study using collaborative causal models. *Futures*, *55*, 58–77. https://doi.org/10.1016/j.futures.2013.11.001

Wächter, P. (2013). The impacts of spatial planning on degrowth. *Sustainability*, *5*(3), 1067–1079. https://doi.org/10.3390/su5031067

Xue, J. (2014). Is eco-village/urban village the future of a degrowth society? An urban planner’s perspective. *Ecological Economics*, *105*, 130–138. https://doi.org/10.1016/j.ecolecon.2014.06.003

1. This review was performed before the most recent COP took place in Sharm El-Sheikh, Egypt (2018) and before IPBES released its global assessment (2019), both of which would otherwise have been considered. [↑](#footnote-ref-1)
2. The COP 13 Final Report was not published when the document selection was conducted. [↑](#footnote-ref-2)
